# Counter pressure maneuvers for syncope prevention: A semi-systematic review and meta-analysis

**DOI:** 10.3389/fcvm.2022.1016420

**Published:** 2022-10-13

**Authors:** Erin Lori Williams, Farhaan Muhammad Khan, Victoria Elizabeth Claydon

**Affiliations:** Cardiovascular Physiology Laboratory, Department of Biomedical Physiology and Kinesiology, Simon Fraser University, Burnaby, BC, Canada

**Keywords:** syncope, orthostatic intolerance, postural sway, counter pressure maneuvers, blood pressure, cardiovascular control, skeletal muscle pump

## Abstract

Physical counter pressure maneuvers (CPM) are movements that are recommended to delay or prevent syncope (fainting) by recruiting the skeletal muscle pump to augment cardiovascular control. However, these recommendations are largely based on theoretical benefit, with limited data evaluating the efficacy of CPM to prevent syncope in the real-world setting. We conducted a semi-systematic literature review and meta-analysis to assess CPM efficacy, identify literature gaps, and highlight future research needs. Articles were identified through a literature search (PubMed, April 2022) of peer-reviewed publications evaluating the use of counter pressure or other lower body maneuvers to prevent syncope. Two team members independently screened records for inclusion and extracted data. From 476 unique records identified by the search, 45 met inclusion criteria. Articles considered various syncopal conditions (vasovagal = 12, orthostatic hypotension = 8, postural orthostatic tachycardia syndrome = 1, familial dysautonomia = 2, spinal cord injury = 1, blood donation = 10, healthy controls = 11). Maneuvers assessed included hand gripping, leg fidgeting, stepping, tiptoeing, marching, calf raises, postural sway, tensing (upper, lower, whole body), leg crossing, squatting, “crash” position, and bending foreword. CPM were assessed in laboratory-based studies (*N* = 28), the community setting (*N* = 4), both laboratory and community settings (*N* = 3), and during blood donation (*N* = 10). CPM improved standing systolic blood pressure (+ 14.8 ± 0.6 mmHg, *p* < 0.001) and heart rate (+ 1.4 ± 0.5 bpm, *p* = 0.006), however, responses of total peripheral resistance, stroke volume, or cerebral blood flow were not widely documented. Most patients experienced symptom improvement following CPM use (laboratory: 60 ± 4%, community: 72 ± 9%). The most prominent barrier to employing CPM in daily living was the inability to recognize an impending faint. Patterns of postural sway may also recruit the skeletal muscle pump to enhance cardiovascular control, and its potential as a discrete, proactive CPM needs further evaluation. Physical CPM were successful in improving syncopal symptoms and producing cardiovascular responses that may bolster against syncope; however, practical limitations may restrict applicability for use in daily living.

## Introduction

Syncope (fainting; a transient loss of consciousness due to inadequate cerebral perfusion) (1) is common; the cumulative lifetime incidence of syncope is estimated to be greater than 35% in the general population (2), although the true prevalence rate is hard to identify as episodes are often under-reported in the general public (3). The age distribution for syncope is bimodal, with episodes most commonly observed in adolescents and the elderly (2). Syncope accounts for up to 2% of emergency department visits, of which 12–85% of patients are subsequently admitted to the hospital, with admission rates varying depending on the country and respective healthcare system considered (4). This presents a substantial healthcare burden, with an annual cost to the healthcare system of up to $2.4 billion annually in the United States (5, 6).

Up to 30% of those who have fainted will experience recurrent and severe episodes (7). Living with recurrent syncope presents many challenges to daily living, and can profoundly impact patient independence, and quality of life (8–12). Patients with syncope also face greater fatigue in daily living (13), and an increased risk of fall-related injury associated with their loss of consciousness, particularly in older adults with increased frailty (14, 15). Up to 30% of patients with syncope will experience injury or physical trauma secondary to their loss of consciousness (16, 17) with approximately 5% of individuals experiencing a significant injury that leads to further impairments in quality of life (18). This heightened risk increases both morbidity and mortality in older adults with syncope (19).

**FIGURE 1:**
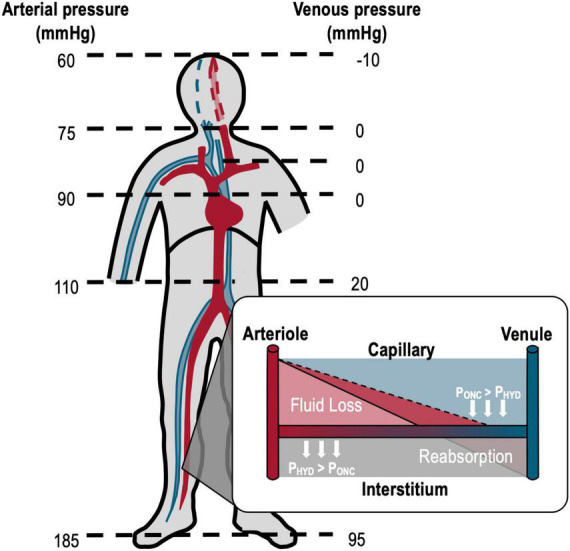
Effects of gravity on arterial and venous pressures in an upright, motionless human. Orthostatic arterial and venous pressures are shown on the left and right, respectively. With orthostasis, arterial and venous pressures above heart level decrease while pressures below the heart increase due to the gravitational redistribution of blood (venous pooling). The white inset diagram shows Starling forces of hydrostatic pressure (P_HYD_, light red shading; generated by the circulating blood pressure, which force fluid out of the vessel) and oncotic pressure (P_ONC_, solid line; generated by plasma proteins, which promote fluid reabsorption) at the capillaries. In orthostasis, the increased lower extremity hydrostatic pressure (dark red shading, dashed line) promotes fluid loss at the capillaries and thus the accumulation of edema in the tissue. The combined venous pooling and capillary filtration reduce the effective circulating blood volume compromising cardiovascular control. Adapted from Hainsworth et al. (78).

The final common pathway for syncope is a reduction in cerebral blood flow, which is exacerbated by orthostatic or gravitational stress, and as such syncope is typically associated with prolonged standing. When upright, gravitational fluid shifts redistribute blood into the lower limbs (venous pooling), which promotes capillary filtration and subsequent edema (Figure 1) (20–23). This effect can be substantial; as much as 700 ml of fluid will accumulate in the lower body with just 10 min of 60° head upright tilting (22, 24). As fluid is lost to the periphery, there will be a concordant decrease in effective circulating volume, leading to decreased blood pressure and venous return, with reductions in cardiac output (CO) that ultimately reduce cerebral perfusion (25, 26). This reduction in blood pressure initiates baroreflex-mediated vascular resistance and capacitance responses, combined with increases in heart rate and force of contraction, to increase blood pressure and regain circulatory homeostasis (27). However, in the presence of a sufficiently severe orthostatic stress, the baroreflex response may be overwhelmed, with the responses evoked insufficient to regain cardiovascular homeostasis. This can result in marked cerebral hypoperfusion, often associated with symptoms of presyncope, and ultimately, syncope (20, 21, 28). In many types of recurrent syncope, these reflex responses may be impaired or compromised, further predisposing to syncopal events. This is most notable with neurally-mediated syncope (vasovagal), postural orthostatic tachycardia syndrome (POTS), or neurological causes of syncope (e.g., autonomic failure) (29).

**FIGURE 2:**
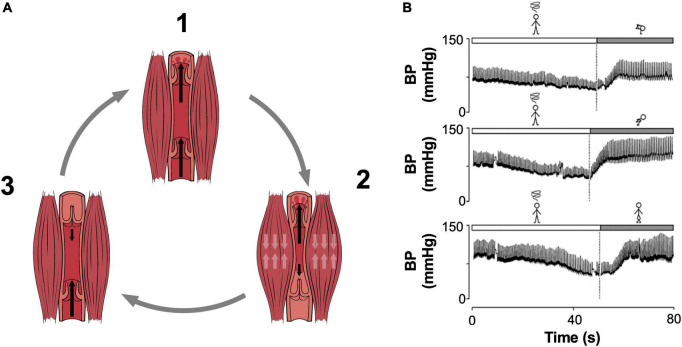
The Skeletal Muscle Pump. **(A)** Pumping cycle of the skeletal muscle pump is shown. (1) Prior to contraction, venous valves are open, and blood freely enters the vessel. (2) Muscular contraction compresses the vessel, forcing blood through the superior valves, meanwhile inferior valves close to prevent backflow. (3) With relaxation, negative pressure closes the superior valves and blood is drawn into the vessel through the inferior valve in preparation for the next cycle. **(B)** Representative sample tracings of pressure responses to the crash position (top; squatting with the head placed between the knees), squatting (middle), and leg crossing performed with buttock and abdominal tensing (bottom). The patient performed these maneuvers following a vasovagal reaction accompanied by prodromal pallor and sweating. All three maneuvers successfully restored blood pressure. BP, blood pressure. Adapted from **(A)** OpenStax College, *Anatomy and Physiology* (133) (licensed under the Creative Commons Attribution 3.0 Unreported license) and Boron and Boulpaep, *Medical Physiology* (27), **(B)** Krediet et al. (134).

The skeletal muscle pump (Figure 2) also helps to counter the effects of orthostatic stress, by facilitating the ejection of pooled blood from the lower extremities back to the heart, and by aiding fluid movement through lymphatic vessels. This pump incorporates any lower body musculature (abdominal, gluteal, thigh, calf) with a role in protecting central blood volume. When recruited, their contraction increases intramuscular pressure to mechanically compress nearby veins, propelling any pooled blood past one-way valves to improve venous return, and subsequently, CO. The benefit of this pumping mechanism in cardiovascular control is clear; populations unable to recruit the skeletal muscle pump, such as patients with paraplegia or tetraplegia, experience increased susceptibility to postural hypotension that can be reversed with electrically stimulated contractions of the lower limb (30, 31). Further, the pumping action induced by intermittent calf compression (32, 33), but not sustained calf compression (34), delays the onset of presyncope and prevents venous pooling in healthy individuals.

Physical counter pressure maneuvers (CPM) are movements that promote cardiovascular stability and boost blood pressure through exploitation of the skeletal muscle pump, often accompanied by increases in sympathetic drive (1, 35–37). Movements such as leg crossing, arm tensing, and squatting are a commonly recommended component of syncope management for patients with recurrent fainting (29, 38), or those prone to blood-injection-injury phobia (e.g., vaccination, blood draws, blood donation) (39–41). Other finer lower body movements such as postural sway have also been linked to fluid volume shifts (42), while enhanced postural sway has been detected in populations susceptible to fainting such as Alzheimer’s disease (43), or astronauts after prolonged space flight (44). At the onset of presyncope, (near-fainting; marked by prodromal symptoms such as light headedness, “cold” sweating, nausea, visual tunneling, and disorientation) (29, 45, 46), patients are often advised to perform CPM to prevent the progression of their symptoms into a frank syncopal episode. Reports indicate that 79–95% of patients with vasovagal syncope (VVS; the “common” faint) experience some form of prodrome (37, 47–49), and 69% feel they are able to identify an impending faint (37). This may permit the deployment of CPM to act as a final line of defense against loss of consciousness once cardiovascular instability is apparent.

While CPM are often recommended to patients, these are based largely on clinical experience with a paucity of direct evidence for their benefit. Systematic reviews performed by Dockx et al. (50), and Jensen et al. (51), provided an important assessment of the quality of evidence to date, with both concluding that the evidence for CPM is of low, or very low quality. In addition, these systematic reviews employed strict inclusion criteria, which aids comparison of similar studies, but at the cost of limited scope, and accordingly, both considered only a narrow selection of articles. To date, full consideration of the responses to CPM and their efficacy is still an emerging field, and comprehensive evaluation of the heart rate (HR) responses to CPM has not been performed; this is of particular interest to patients with POTS who experience debilitating symptoms of orthostatic tachycardia associated with presyncopal symptoms. Further, responses of the cerebral circulation to CPM have not been evaluated; cerebral hypoperfusion is the final common pathway for syncope, therefore identifying cerebral responses is key to understanding the potential of CPM to provide a benefit for syncope prevention. Finally, prior works have focused on CPM that are most commonly recommended by clinicians. While this is a key area of research interest, there is also value in considering non-traditional maneuvers (e.g., neck flexion, seated maneuvers, marching, calf raises, abdominal compression, postural sway) in discussing future research needs as the field progresses. A more exhaustive summary of the diverse investigations performed to date is therefore needed in order to adequately consolidate current understanding of the utility of CPM for syncope management.

## Aims and scope

In the present review, we take a semi-systematic approach to exploring the utility of CPM in syncope prevention. First, we provide an overview of the cardiovascular responses to these movements and explore plausible mechanisms for their utility. We also review the evidence for the efficacy and practical applicability of CPM that are currently recommended. Finally, we explore the role of postural sway in the maintenance of cardiovascular homeostasis, as this movement may provide a discrete, versatile, and accessible adjunct to currently recommended CPM.

## Methodology

A semi-systematic approach was used to retrieve and review the current literature, as this is recommended for topics that have been conceptualized differently by various groups of researchers within diverse disciplines (52). This methodology uses the unbiased and rigorous search technique of a systematic review to identify a pool of literature, while allowing for flexibility in the dissemination of findings. Of note, a semi-systematic method permits the narrative review of emerging themes and knowledge gaps, and can highlight the complementary and contrasting techniques that have been used to study a given topic (53).

**FIGURE 3:**
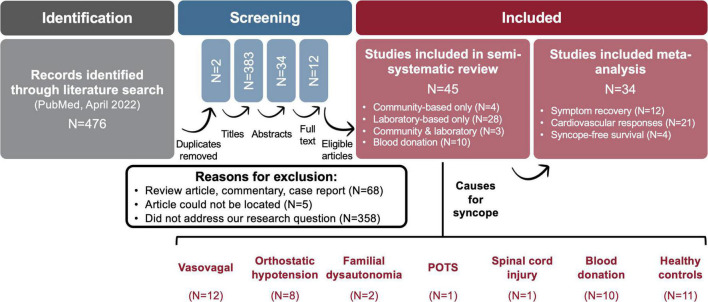
Semi-systematic method for literature review. The above flow diagram depicts our article selection process. Articles were screened at various levels (titles, abstracts, full text) to identify those that were relevant. Works were included if they evaluated the role of counter pressure and other lower body maneuvers in preventing syncope. Articles were selected for meta-analysis based on common themes amongst the article pool. Articles identified for our review that did not evaluate outcomes considered for meta-analysis were discussed in narrative form. POTS, postural orthostatic tachycardia syndrome.

In our literature search, we identified works evaluating the role of CPM and other lower body maneuvers in preventing syncope (Figure 3). The initial search was conducted in the MEDLINE (PubMed) database (April 28, 2022) using the pre-determined search terms, and two reviewers (E. L. W. and F. M. K.) screened results using agreed-upon inclusion/exclusion criteria (Table 1). Our search originally uncovered 476 articles, and through the systematic screening of titles, abstracts, and full-text articles, 45 works were selected for inclusion. Articles with common themes were included in various meta-analyses (*N* = 34), while articles that did not evaluate these common outcomes were discussed in narrative form (*N* = 45).

**TABLE 1:** Semi-systematic search strategy.

Database	Search Terms	Inclusion Criteria	Exclusion Criteria
MEDLINE (PubMed)	[(syncope) OR (orthostatic tolerance) OR (postural syncope) OR (autonomic failure) OR (sympathetic failure) OR (orthostatic hypotension) OR (lower body negative pressure)] AND [(postural sway) OR (muscle pump) OR (counter pressure) OR (counter pressure) OR (leg crossing) OR (tensing) OR (muscle tension) OR (squatting)]	Investigated cardiovascular, cerebrovascular, or other physiological responses to CPM. Investigated the efficacy of a given CPM to reduce symptoms of presyncope or presyncopal events or contribute to recurrent syncope management more broadly (e.g., improve quality of life). Investigated underlying mechanisms for the ability of muscular movement recruiting the skeletal muscle pump to contribute to cardiovascular and/or cerebrovascular stability. CPM defined as voluntary movements, either sustained or repetitive, performed for the purpose of maintaining or regaining cardiovascular stability.	Used body movement as a means to induce presyncope (e.g., rapid squat to stand) for the sake of evaluating a different (non-CPM) intervention. Evaluated CPM in combination with additional treatments (outside those typically prescribed as part of usual care) that may also provide a benefit in syncope management. Movements were not considered as CPM if they were: 1. better classified as exercise (e.g., resistance or cardiovascular training); 2. required external equipment (e.g., passive movement, external compression); or 3. were not performed voluntarily (e.g., electrical stimulation).

Agreed upon search terms, inclusion, and exclusion criteria for our systematic article search are reported above. CPM, counter pressure maneuver.

## Counter pressure maneuvers

### Cardiovascular responses

Cardiovascular responses to CPM have been studied most widely, with 21 articles considering responses in the laboratory setting (30, 36, 54–73). A summary of the cerebrovascular and hemodynamic responses to various CPM is reported in Table 2. While the movements performed during CPM constitute a small exercise dose, these may still be sufficient to initiate a physiological exercise response concurrent with any muscle-pumping effect (36, 54). In addition, while most CPM involve an isometric (sustained) contraction, some require dynamic movements with oscillating contraction and relaxation of major muscle groups. In our literature pool, nearly all maneuvers were sustained, however, cardiovascular responses to dynamic movements (tiptoeing and marching) were considered in two studies (60, 64). Further investigation into dynamic maneuvers would improve our understanding of these CPM and how they might compare to sustained movements, particularly since rhythmic contraction/relaxation could be easier to maintain and may better facilitate muscle pumping activity (32, 33, 74, 75).

**TABLE 2:** Cardiovascular responses to counter pressure maneuvers.

Maneuver	Population	ΣN	SAP mmHg	DAP mmHg	MAP mmHg	CO L.min^–1^	HR Bpm	SV %	TPR %	CBFv cm.s^–1^
Neck flexion	OH (64)	9	↑							
Upper limb tensing	Control (58, 67, 73)	80	↑↑↑ –	↑↑ –		↑↑↑	↑ –	↑		
	VVS (67, 73)	69	↑ – –	↑↑ –		–	– –			
Abdominal contraction	Control (94)	10	–	–			↑			
	OH (64)	9	–							
	FD (62, 94)	23	↑ –	–	↑	↑	– ↑		–	
Lower body tensing	Control (55, 58)	13	↑		↑↑	↑ –	↑	↑	↑↓	
	VVS (36, 56)	30			↑↑	↑↑	↑	↑	– –	
	OH (55, 64)	22	↑		↑	–			–	
Leg crossing	Control (54, 60, 65, 71)	27	– –*	– –*	– – –	↑ – –	↑↓↓	↑↑↑	– – –	↑
	VVS (63)	88	↑	↑	↑	↑	–	↑	↓	
	OH (60, 64, 65, 71)	29	↑↑↑*	↑↑*	↑↑↑*	↑↑	– –	↑↑	↑–	↑
	FD (62)	12	↑	↑	–	–	–		–	
Leg crossing (seated)	Control (69)	43	↑	↑			↑			
Leg crossing and tensing	Control (61, 66, 70, 73)	54	↑	–	↑↑ –	↑	↓↓ –			↑
	VVS (36, 63, 72, 73)	137	↑↑↑↑	↑↑↑ –	↑↑	↑↑	↑↑↓ – –	↑↑	↓ –	
	OH (61, 97)	34			↑↑	–	↓	↑	–	↑
Leg crossing and tensing (seated)	Control (69)	43	↑	↑			↑			
Tiptoeing	Control (60)	6	↑	–	↑	↑	–	↑	↓	
	OH (60)	7	–	–	–	↑	–	↑	↓	
Marching	OH (64)	9	↑							
Calf raises	OH (64)	9	↑							
Leg fidgeting	OH (68)	6	↑*	↑*	↑*					
Kneeling	OH (64)	9	↑							
Squatting	Control (65, 73)	27	↑↑*	↑–*			–			
	VVS (36, 73)	64	↑↑	↑↑	↑	↑	– – –	↑	–	
	OH (64, 65)	16	↑↑*	↑*	↑*					
	FD (62)	12	↑	↑	↑	↑	–		↑	
Bending Forward	FD (62)	13	↑	↑	↑	↑	–			
Whole body tensing	VVS (36)	9			↑	↑	–	↑	–	
Crash position	VVS (36)	9			↑	↑	↓	↑	–	

The assessments summarized considered healthy controls (Control), as well as patients with vasovagal syncope (VVS), orthostatic hypotension (OH: including initial OH, classical OH, and hypoadrenergic OH secondary to autonomic failure), and familial dysautonomia (FD). Each entry denotes reports from a single study, with increases (↑) or decreases (↓) in physiological outcome measures reported where significance criteria of p < 0.05 were met. The horizontal line (–) indicates that no significant change in the outcome measure was detected. Blank spaces indicate that the outcome was not considered by a given study. Note that where an individual study performed more than one evaluation of a counter pressure maneuver, or provided analyses across multiple subgroups of patients or controls, more than one outcome was generated. Upper limb tensing includes both arm tensing, and hand grip maneuvers. Lower body tensing includes instruction to contract any combination of musculature of the lower body (abdomen, buttocks, thigh, calf, entire leg) without any additional instruction for movement or position change. *Significance was determined by student’s t-test using data reported in publication. SAP, systolic arterial pressure; DAP, diastolic arterial pressure; MAP, mean arterial pressure, CO, cardiac output; HR, heart rate; SV, stroke volume; TPR, total peripheral resistance (measures retrieved included total peripheral resistance, systemic vascular resistance, and peripheral vascular resistance); CBFv, cerebral blood flow velocity; ΣN, cumulative sample size.

**FIGURE 4:**
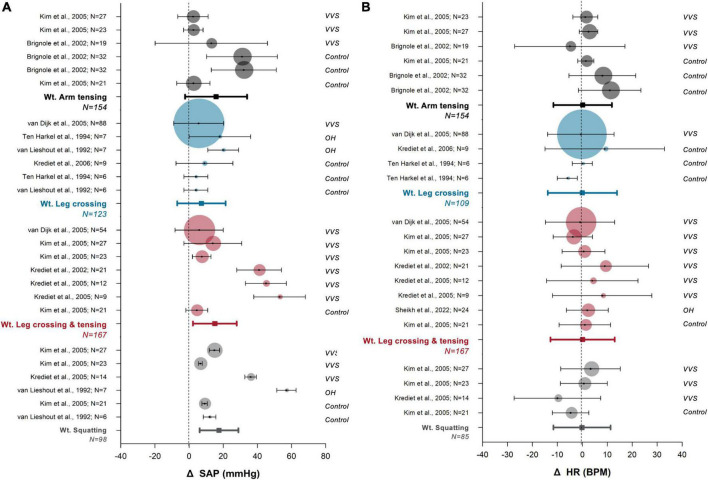
Meta-analysis of cardiovascular responses to commonly prescribed counter pressure maneuvers. Responses of systolic arterial pressure [SAP, **(A)**] and heart rate [HR, **(B)**] are shown. The most highly studied counter pressure maneuvers were arm tensing (black), leg crossing (blue), leg crossing with muscle tensing (red), and squatting (gray). Data for each study are presented as the mean (circle) and standard deviation (whiskers), with the size of the circle proportional to the study sample size. Bolded whisker plots show the weighted (wt.) mean and standard deviation of group analyses. Vertical dotted lines indicate zero effect. The population considered is indicated to the right of each plot: vasovagal syncope (VVS), orthostatic hypotension (OH, included classical OH, and hypoadrenergic OH), and healthy controls (control).

Most studies reported that CPM were successful in improving both systolic (SAP) and diastolic (DAP) arterial pressure, as expected during sustained CPM. Isometric muscle contraction leads to concurrent increases in SAP and DAP, driven by a net increase in total peripheral resistance (TPR) which occurs largely due to the mechanical compression of the vasculature by working muscle (36, 76, 77). Following tiptoeing (a dynamic maneuver), an increase in SAP with no change in DAP was reported, which is expected during dynamic exercise. In our combined analysis of SAP responses to the most commonly studied CPM (Figure 4A) SAP increased by + 14.8 ± 0.6 mmHg (*N* = 628, *p* < 0.0001), demonstrating evidence of benefit of CPM for syncope prevention.

A small but significant HR response to CPM was detected (+ 1.4 ± 0.5 bpm; *N* = 539, *p* = 0.006) (Figure 4B) and this may reflect contradicting regulatory mechanisms. Baroreflex-mediated bradycardia would be expected in response to recovered blood pressure, and this would be augmented by increased stroke volume following increased preload (54). However, any concurrent exercise response caused by CPM would lead to increased HR (36, 78, 79). The combination of both stimuli likely explains why we detected only a small net effect on the HR response.

In almost all studies that reported CO responses, CO was increased following CPM. Given the small HR response to CPM identified in our combined analysis, this suggests that improvements in CO are likely driven by the well documented increase in SV (observed in every study that examined SV responses), reflecting improved venous return, and ultimately, effective skeletal muscle pumping (1, 35, 36). Due to a lack of uniformity in reporting methods for SV responses (with different units, and variations in reporting of absolute responses, absolute changes, or percentage changes) we were unable to perform meta-analyses on SV data.

TPR responses during sustained (isometric) CPM were variable, with some studies reporting increases and some reporting decreases in TPR. This likely also reflects contradictory regulatory mechanisms with different CPM approaches. As with SV data, a combined analysis on TPR was not possible due to a lack of uniformity in reporting measures. During exercise, muscle mechanoreflexes and chemoreflexes promote increased TPR through increased sympathetic nerve activity. Of note, chemoreflex responses may not contribute to all findings reported, as they only become prominent after approximately 1 min of sustained muscular contraction (36, 63, 79). The mechanical compression of vasculature by sustained isometric contraction also contributes to increased TPR (36, 76, 77), but this may be countered by baroreflex-mediated decreases in TPR as blood pressure recovers due to successful skeletal muscle pumping. Interestingly, in patient populations with reduced sympathetic drive (e.g., pure autonomic failure, multiple system atrophy), the mechanical increases in TPR may be even more beneficial by countering the vasodilatory effects caused by reduced sympathetic vascular control (71). In one study that evaluated dynamic CPM (tiptoeing), a net decrease in TPR was reported as expected. Further investigation is required in both sustained, and dynamic CPM to clarify the TPR response, and tease apart the many regulatory mechanisms at play.

### Cerebrovascular responses

The final common pathway for syncope is a reduction in cerebral perfusion (80, 81), therefore cerebral blood flow is a primary outcome of interest; however, cerebrovascular responses to CPM have not been studied widely.

Both cerebral blood flow velocity (CBFv) (61, 66, 71, 82, 83) and cerebral oxygenation (66, 69, 71) are reported to increase following sustained lower body CPM. This likely reflects improved cerebral perfusion in light of the increases in blood pressure, SV and CO that accompany CPM (66, 71). However, one study also reported that bolstering of CBFv with CPM occurred alongside increased P_a_CO_2_ and P_ET_CO_2_ [and therefore ameliorated the orthostatic hypocapnia-mediated cerebral vasoconstriction (66)]. This may be attributable to an exercise response increasing carbon dioxide production (84), or perhaps to reduced reliance on the respiratory muscle pump when cerebrovascular control is bolstered by CPM, reducing postural hyperventilation and associated hypocapnia.

### Treatment efficacy

**FIGURE 5:**
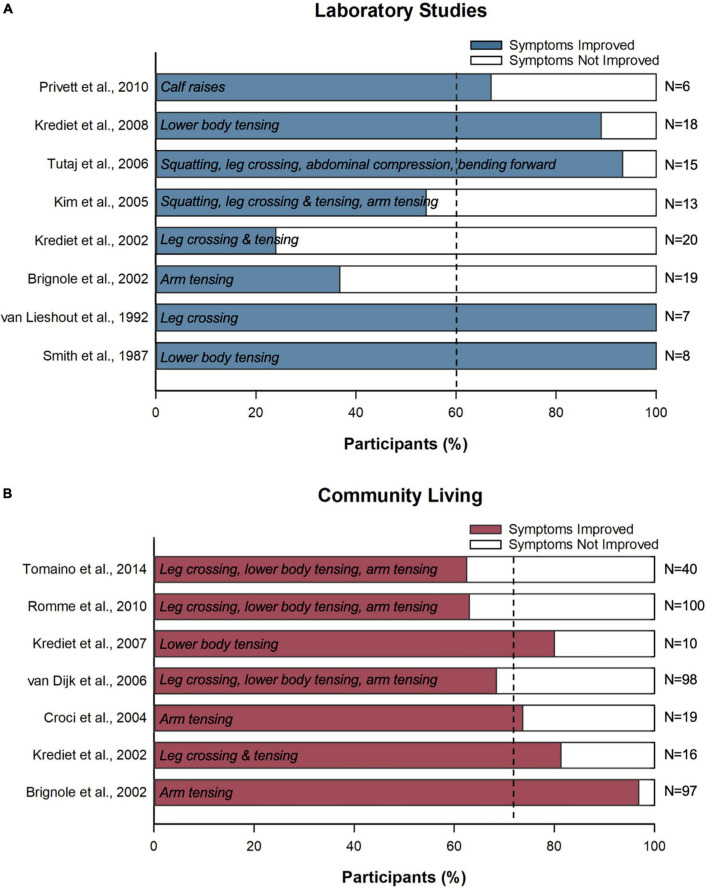
Effects of counter pressure maneuvers on symptoms of syncope and presyncope. Bars show the proportion of patients (%) who reported recovered symptoms following counter pressure maneuver implementation in **(A)** the laboratory and **(B)** the community setting. White text indicates the counter pressure maneuvers investigated in each study. Vertical dotted lines indicate the pooled mean.

CPM are considered a low-cost, first-line management strategy for syncope prevention (35, 36, 65, 67, 72, 73, 85). The European Society of Cardiology guidelines for the diagnosis and treatment of syncope list CPM as a Class I intervention for patients with reflex syncope if they typically experience a prodrome prior to an episode (29). These maneuvers are recommended alongside mainstay treatments of syncope of patient education and lifestyle and management advice (29, 38). They may also provide additional benefit alongside other preventative measures such as the avoidance of known triggers for syncope, blood volume expansion by means of raised water and salt intake, and adjustment of aggravating medications (37, 38, 86, 87). Current CPM recommendations are based primarily on physiological evidence and clinical experience (29, 38), however, clear evidence for the efficacy of these maneuvers in mitigating symptoms is lacking. While CPM are a low-cost prevention strategy with a minimal risk of adverse side effects, an accurate evaluation of efficacy is needed to properly prescribe this treatment, identify populations most likely to benefit, and inform best practice. In our literature pool, we identified 13 studies where symptom recovery following CPM use in combination with usual care was evaluated through patient reports of sensations of presyncope, or the occurrence of a syncopal event (Figure 5).

Our combined analysis revealed that CPM diminished symptoms of presyncope and syncope for 62 ± 4% of patients in the laboratory, and 72 ± 9% of patients when used in combination with usual care during activities of daily living (Table 3). Alizadeh et al. also reported improvement in 65% of patients with use of hand grip, and 75% with use of squatting (compared to 43% with the control treatment of increased hydration and salt consumption). While this study was not identified in our original search, it does align with the findings of our meta-analysis (88). During activities of daily living, while many patients do appear to experience relief from CPM use, the current findings are limited by a lack of control or placebo condition (55, 65, 67, 72, 89), and a small sample size (57, 58, 65). As such, it was difficult to account for episodes of presyncope that would have resolved spontaneously even without CPM use, or the improvement in syncopal events reported relative to the patient’s baseline, or over and above improvements associated with usual care. This is important because, based on follow-up studies that included a control group, 48% of patients experienced improvement in syncopal episodes with usual care alone, compared to 65% experiencing improvement with usual care and CPM use (35, 90). The relatively high improvement rates in the control arm make the efficacy of CPM harder to ascertain and may reflect regression to the mean, improvements in syncopal symptoms in response to standard care and lifestyle advice, as well as the benefit of patient education or the relief associated with receiving diagnostic testing or an adequate diagnosis, all of which are known to contribute to improved patient outcomes (91, 92). A strong placebo effect has also been noted in syncope research, with patient expectancy for improvement possibly playing a further role in episode reduction (93). While this highlights the need for placebo controlled studies, it also suggests that perhaps the reassurance and confidence that accompanies training with a “safety-net” type strategy such as CPM may contribute to a positive benefit (35).

**TABLE 3:** Improvement of symptoms of syncope and presyncope with counter pressure maneuvers.

Paper	N	Population	Setting	Follow Up (Mo)	Endpoint	Description	Maneuver	Improvement (%)
Privett et al., (57)	6	Control	Post-exercise	–	OI Reoccurrence	Control: 6/6 (100%) had OI CPM: 2/6 (33%) had OI	Calf raises	67
Krediet et al., (56)	13	VVS	Sit-stand	–	Presyncope	Control: 13/18 (72%) reported presyncope CPM: 2/18 (11%) reported presyncope	Lower body tensing	89
Tutaj et al., (62)	15	FD	Standing	–	Presyncope	14/15 (93%) verbally reported reduced symptoms with CPM	Squat, leg cross, abdominal compression, bend forward	93
Kim et al., (73)	13	VVS	Tilt (Passive)	–	Tilt Endpoint	Control tilt: 13/13 (100%) positive Tilt + CPM: 6/13 (46%) positive	Leg crossing	54
Brignole et al., (67)	19	VVS	Tilt (Passive)	–	Tilt Endpoint	Control tilt: 19/19 (100%) positive Tilt + CPM: 12/19 (63%) positive	Hand grip	37
Krediet et al., (72)	21	VVS	Tilt (Passive)	–	Tilt Endpoint	Reaction averted with CPM in 5/21 patients (24%), 15/21 (71%) had delayed syncope by median 2.5 min (30 s–11 min)	Leg crossing and tensing	24
van Lieshout et al., (65)	5	OH	Sit-stand	–	Presyncope	Control: 5/5 (100%) reported presyncope Stand + CPM: 0/5 (0%) reported presyncope	Leg crossing	100
Smith et al., (58)	8	Control	Tilt (LBNP)	–	Tilt Endpoint	Control tilt: 6/8 (75%) positive Tilt + CPM: 0/8 (0%) positive	Lower body tensing	100

							**Weighted Mean:**	**62 ± 4**

Tomaino et al., (90)	95	VVS	Community	16	Syncopal Reoccurrence	Reoccurred in 15/40 (37%, CPM), 24/45 (53%, control at follow up	Leg crossing, lower body tensing, arm tensing	63
Romme et al., (96)	100	VVS	Community	12	Syncopal Reoccurrence	63/100 (63%) using CPM experienced a decreased syncope burden (episodes/year) at follow up	Squatting, leg-crossing, lower body tensing, arm tensing	63
Krediet et al., (36)	10	OH	Community	2	Presyncope	8/10 (80%) patients reported reduced symptoms with CPM	Lower body tensing	80
van Dijk et al., (35)	85	VVS	Community	12	Syncopal Reoccurrence	Reoccurred in 31/98 (32%, CPM), 56/110 (51%, control) at follow up	Leg crossing, lower body tensing, arm tensing	68
Croci et al., (89)	19	VVS	Community	14	Syncopal Reoccurrence	5/29 (17%) patients experienced syncopal reoccurrence at follow up	Arm tensing	74
Krediet et al., (72)	16	VVS	Community	10	Syncopal Reoccurrence	13/16 (81%) with recurrence experienced fewer episodes than in previous year	Leg crossing and tense	81
Brignole et al., (67)*	97	VVS	Community	9	Presyncope	Among 11 patients (median 3 syncopal episodes/year), 94/97 (97%) cases of presyncope resolved.	Arm tensing	97
							**Weighted Mean:**	**72 ± 9**

Reports from studies evaluating counter pressure maneuvers (CPM) in the laboratory (top) and community setting (bottom, employment of counter pressure maneuvers in daily living) are summarized. The weighted mean **±** standard deviation (%) of participants improved is reported under each respective list. These assessments considered populations of healthy controls (control), as well as patients with vasovagal syncope (VVS), orthostatic hypotension (OH, included initial OH and classical OH), and familial dysautonomia (FD). The follow up periods of community-based studies are given in months (Mo). Improvement was characterized by reports of the onset and subsequent alleviation of symptoms via counter pressure maneuvers, as indicated by the endpoint of the study. The endpoint for improvement varied across studies: OI reoccurrence, symptoms of orthostatic intolerance detected; presyncope, detected with cardiovascular cut-offs or patient reports; tilt endpoint, detected as per experimental protocol; syncopal reoccurrence, episode of syncope reported by patient over follow up period. Individuals that did not experience symptom onset initially or over the trial period were excluded from analyses. Individuals that were unable to employ counter pressure maneuvers for any reason were included in the analysis. Because most studies did not incorporate a control, or crossover design, improvement was calculated based on intervention group only. *Denotes study that used pseudo-replication in reporting of results; episodes of syncope experienced by 11 participants were pooled, and the improvement of individual participants was not evaluated.

There was also a lack of standardized methodology to document patient symptoms, with those used often relying on patient reports or extended recall of events (57, 58, 65). Of note, Brignole et al. (67) evaluated CPM success by pooling total syncopal episodes experienced among a small sample of patients. While this group reports one of the highest success rates (97% recovered), their methods involve pseudo replication, and fail to account for any episodes of presyncope that would not have progressed into syncope even in the absence of deployment of CPM. With this cohort reporting a median of 3 (1–11) syncopal episodes in the prior year, the majority of presyncopal episodes assessed would have presumably resolved spontaneously (67). For a clearer understanding of the efficacy of CPM in managing syncope symptoms, more rigorous and unbiased assessments including a placebo or control treatment are needed.

Following the release of CPM, symptoms may return (55, 56, 71, 73); although complete recovery is most beneficial, delaying symptoms can be valuable in preventing a fall (and related injuries). In some patients that did not experience complete symptom recovery, CPM were still successful in delaying syncope by approximately 2.5 min (67, 72). Further, patients using CPM also experienced quicker cardiovascular recovery when an orthostatic stress was introduced following maneuver release (69, 94). In practice, this could be enough time to assume a more protective position (lying down, sitting down), or call for assistance. Combining CPM can be particularly beneficial in this regard, with one movement serving to dampen symptoms, and the second aborting symptoms altogether (56). For example, squatting produces a large cardiovascular response, and was successful in aborting symptoms while the maneuver was sustained (alongside a practical benefit of being less socially embarrassing than lying down); however, when participants return to the standing position, symptoms often resume (55, 56, 73). Symptom recurrence is common with rising from squatting in particular, due to the removal of relative ischemia (due to mechanical compression during the maneuver, the “post tourniquet effect”) (95) and an abrupt increase in gravitational stress upon standing. Therefore, the duration of time spent in a squat may affect the syncope recurrence (55). Patients have found success in combining the rise from squatting with an additional maneuver (e.g., leg tensing, lower body tensing) to abort the milder presyncope they experienced upon orthostasis. In future investigations, the evaluation of the symptom recurrence following termination of the maneuver would be helpful in better stratifying currently recommended movements, as interventions that most often terminate symptoms at first use are more practical than those that permit a return of presyncope when terminated and must be repeated.

### Practical application

Thus far, much of the documented benefit of CPM comes from laboratory studies, and few studies have included follow-up assessments considering their use in daily living (35, 55, 67, 72, 89, 90, 96). Only four studies have directly evaluated the real-world efficacy of CPM; while two of these provide high level evidence (35, 90), two are limited by a lack of comparator group (89, 96). There is, therefore, a need for more randomized controlled trials considering the efficacy of various CPM in terminating impending syncope.

**FIGURE 6:**
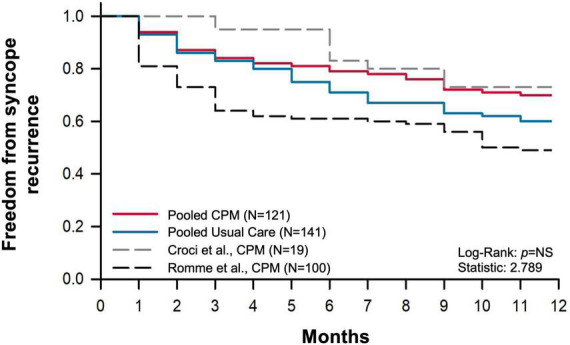
Kaplan–Meier syncope-free survival curve. Pooled time to first syncopal recurrence over a 1-year follow up in groups provided with usual care and trained in counter pressure maneuvers (CPM), and controls (usual care only) were compared (9, 87). Each study followed patients with vasovagal syncope who experienced prodromal symptoms prior to syncope. Data reported by Croci et al. (86) and Romme et al. (93), (dashed) did not include a control group and therefore were not included in combined analysis.

We conducted a combined survival analysis (time to first syncopal recurrence in months) for the available data reporting efficacy of CPM in the community setting (Figure 6). When comparing combined analyses (35, 90) between patients using CPM (*N* = 121) and those treated with usual care (*N* = 141), we found no significant differences in time to first syncopal recurrence with the adoption of CPM (Figure 6). However, the combined relative risk reduction in syncopal occurrence with CPM was 34% (relative risk with CPM: 0.67, CI: 0.51 to 0.88, *p* = 0.004), which was associated with a number needed to treat of 5.75 patients (*p* = 0.004), and an odds ratio of 0.49 (CI: 0.31 to 0.78, *p* = 0.003). Of note, in our combined analyses, we did not include studies that did not incorporate a control group (89, 96) (although they are provided for interest in Figure 6); the findings of Croci et al. were not significantly different from our combined CPM results, but those reported by Romme et al. reported more limited success of CPM, with a significantly shorter time to first syncopal recurrence (*p* = 0.005). The reasons for this discrepancy are unclear; all four studies recruited a similar patient population with a similar frequency of syncopal events prior to enrollment in the study. In summary, as noted previously, there was a clear benefit of CPM on cardiovascular responses during laboratory testing. However, the efficacy of CPM in the community setting is less clear, and this may reflect challenges with adopting CPM in the real-world.

Evaluating practical efficacy is complex, as there are many factors outside physiological benefits at play. In CPM, consideration of prodromal history is of interest, as a patient must be able to recognize and employ CPM effectively prior to losing consciousness in order for them to be effective (72). While most follow-up assessments considered only patients with a significant prodrome (35, 89, 96), approximately 47% of participants assessed by Tomaino et al. (90) had a history of some sudden episodes without presyncope. In their complete analyses, this group did not detect a significant difference in 21-month syncope free survival between CPM and usual care. However, when considering only patients with a recognizable prodrome, although there was still a lack of significant difference in syncope-free survival, relative risk reduction was slightly improved. When evaluating the true efficacy of CPM among all patients, a wider population should be considered to avoid over estimation of benefit, as many patients with syncope do not experience a prodrome (approximately one-third of patients with VVS) and this obviously limits their ability to adopt CPM (48).

In many studies, even those considering only patients with a known history of syncopal prodromes, insufficient time to enable execution of CPM prior to loss of consciousness was a prominent barrier to their use in daily living (35, 55, 72, 89, 90, 96). To address this barrier, discrete and proactive use of maneuvers in provocative situations may improve CPM accessibility to a wider range of patients. Muscle tensing prior to a stand can bolster blood pressure more than tensing after standing and was beneficial in preventing initial orthostatic hypotension; this could perhaps be combined with a classical counter maneuver to provide a further benefit (97). Dynamic maneuvers may also be of particular benefit as they could better facilitate muscle pumping and can be maintained for longer periods of time (as they incorporate cyclical contraction and relaxation) (32, 34, 74, 75). In this case, the maneuvers could be performed proactively when one knows they are in a provocative situation (e.g., for occupations requiring sustained motionless standing, or experiencing known triggers for patients).

Many studies evaluating the practical applicability of CPM are also limited by a lack of a placebo control or comparison to baseline syncope frequency (55, 67, 72, 89). This is especially noteworthy, as the role of CPM in syncope management is not necessarily to eliminate episodes all together. Patient quality of life is inversely related to episode frequency (12), therefore reducing episodes could still lower syncope burden, bringing a substantial benefit to the patient (90, 96). Over a 1-year follow up, Romme et al. reported that while 49% of patients experienced a syncopal recurrence, disease-specific quality of life improved most in patients with the largest reductions in syncope burden (as quantified by episode frequency over time) (96). In future studies, additional metrics beyond time to first syncopal recurrence should be considered to evaluate treatment benefit, especially those evaluating a change in symptom frequency or further investigations into quality of life improvement (90, 96).

Proper CPM training is also imperative to effective execution in daily living (64). Syncope onset can be frightening, and even patients with an identifiable prodrome may experience panic at the moment of presyncope onset, causing them to forget the specific movements, or forget to employ them altogether (35, 96). Particularly in patients with a short prodrome, longer training periods may be required to improve patient comfort and confidence in using CPM independently. Training is often achieved using a combination of instruction, visual cues (demonstration, video), and biofeedback during CPM performance by the patient (35, 89, 96). Additionally, patients also must avoid straining (i.e., Valsalva maneuver) during the maneuver as this increases intrathoracic pressure, thereby reducing blood flow to the thorax (63, 72). Given the importance of prodromal recognition, introducing CPM training following a tilt table test may bring a further benefit, as patients will have a safe opportunity to become familiar with their presyncopal symptoms, and better identify these in daily living (67, 72).

Neurally mediated syncope and orthostatic hypotension are particularly prevalent among elderly individuals (15, 98), and elderly patients with syncope that are both frail and have impaired orthostatic blood pressure control also experience a heightened risk of falling (15). While this population would theoretically benefit from employment of CPM should they become symptomatic, CPM appear to be less effective in older patients for whom mobility and strength may be limited (89, 90). Populations with reduced muscularity may require additional time (and therefore a greater prodromal warning sign) to employ the maneuvers properly, and may therefore require more rigorous training, or additional practice at home (35). Actions such as squatting involve difficult transitions such as lowering one’s center of gravity or returning to an upright position from the ground, which could be challenging for patients with limited mobility, strength, or those that experience presyncopal reoccurrence upon standing (55, 56, 73). While leg crossing with tensing has been suggested as a useful alternative to squatting (as it does not involve bending or crouching), leg crossing does, however, reduce the base of support, which could also predispose a fall and subsequent injury in patients with limited balance (73). Patient populations with severe orthostatic hypotension and associated impairments in postural control may face similar difficulties accompanying more challenging movements such as squatting, leg crossing, or bending forward (62). Given the range of complications that could arise in vulnerable populations, it is advisable to teach patients several different CPM, allowing the patient to decide which is the best fit relative to their comfort, mobility, balance, and needs in specific social situations (62).

### Ideal population

CPM have been studied most widely in patients in whom their syncope episodes present with a prominent vascular component; this includes VVS (36, 56, 63, 67, 72, 73), initial orthostatic hypotension (55, 97), and populations with neurological causes for syncope such as autonomic failure (60, 61, 64, 65, 68, 71, 96) or familial dysautonomia (62, 94). Of note, community-based trials documenting syncope free survival with CPM have only been performed in VVS (35, 89, 90, 96). To our knowledge, CPM have not been widely considered for cardiac causes of syncope (e.g., arrhythmia, structural abnormalities), presumably because improvements in venous return would have a limited benefit with this pathology. However, there may be a particular benefit for patients with VVS that are fitted with a pacemaker (90). Cardiac pacing for uncomplicated VVS is controversial but is sometimes employed as a last resort for syncope that is refractory to other treatments, accompanied by severe symptoms, and associated with asystole. However, as many as one-quarter of patients with syncope that also have an implanted pacemaker continue to experience recurrent episodes (90, 99), presumably because the pacemaker does not address the vasodepressor component of their syncope; in these patients CPM may be of benefit to combat this hypotensive component of their episodes. However, this needs to be investigated further as patients with asystolic faints often have minimal prodrome (99), and the complexity of a mixed pathology may reduce the ability of CPM to adequately recover cerebral perfusion and blood pressure (35).

The benefit of CPM in pediatric syncope or POTS has also not yet been widely evaluated, with the latter perhaps also driven by the lack of evidence for meaningful benefit of CPM on orthostatic HR responses. Static hand grip was reported to terminate symptoms in patients aged 15–22 years with initial orthostatic hypotension, however, no further investigations in pediatric patients have been performed to date (51, 100). Neuropathic or “partial dysautonomic” POTS is the most commonly diagnosed subtype and occurs due to an inability to vasoconstrict peripheral vasculature upon standing (101, 102). In response, as blood pools in the lower extremities, excessive increases in HR occur in order to maintain CO and cerebral perfusion (102, 103). Since CPM can reduce venous pooling, and supplement venous return through initiating muscle pumping, it may be that CPM could dampen orthostatic tachycardia in some forms of POTS. This is supported by the observation that some patients with POTS have diminished calf venous capacity, circumference and ejection fraction (59); while the etiology of this observation is still unknown, it does support a role for targeting improvements in skeletal muscle pumping in this population.

Ultimately, more extensive investigation considering CPM use in a wider variety of syncope patients, including both POTS and cardioinhibitory forms of VVS, is needed to better understand the applicability of this intervention, and inform clinical practice. Additionally, evaluations in both adult and pediatric populations should be performed, as children may not respond in the same manner as adults, given their complex physiology and common occurrence of “dysautonomia of adolescence,” dysregulation of the autonomic nervous system during puberty (11).

CPM have also been evaluated for use during syncope associated with blood-injection-injury stimuli, especially during blood donation and intravenous catheter insertion. Multiple studies report that CPM are effective at reducing vasovagal reactions and presyncopal symptoms in this setting (104–110). Responses were more favorable when tensing was applied specifically when the needle was inserted, when the needle was removed, and when getting up from the chair after the procedure (106). Thus far, muscle tensing of the calf, thigh, abdomen, buttock, arm, and chest have been considered (104–113). Applying CPM during or immediately preceding blood donation significantly increases HR (105, 111) and CBFv (111), although responses may vary in patients with heightened anxiety, or those that experience blood-injection-injury phobia (105). Interestingly, when simulated blood draws were performed on a cohort of individuals that were highly fearful of needles, CPM were still effective in producing cerebrovascular benefits (104). Donors using CPM reported greater feelings of confidence and control during the procedure (106), and reduced anxiety (105). Blood donor rate was also greater in those using CPM, particularly for women (112), or those who had a pre-disposing fear to needles (40). Accordingly, CPM appear to be a simple and effective intervention to reduce vasovagal reactions and improve patient comfort during invasive procedures.

## Postural sway

### Sway as a counter pressure maneuver

Through our literature search, a small subset (*N* = 6) of papers were retrieved providing preliminary evidence for a potential role of postural sway as a CPM (114–119). Postural sway describes the fine, unconscious movements that are generated in response to neural feedback in order to maintain balance in the upright position (120, 121). Throughout this process, the musculature of the lower limbs and torso initiate stochastic movements of the body’s many segments to ultimately keep the center of mass [average position of all parts of a system, weighted according to their mass (122)] in motion, within the bounds of their base of support [the area beneath an object or person, encompassed by every point of contact between them and their supporting surface (123)] (124). As the center of mass moves, its two dimensional path within this base can be traced and reduced to movement in the anteroposterior (predominantly controlled by muscles of the calf) and mediolateral (predominantly controlled by muscles of the thigh) directions (125).

The fine movements initiated by postural sway, while small, recruit lower body musculature and may be sufficient to bolster against syncope through skeletal muscle pumping. The oscillatory nature of postural sway would yield benefits in comparison to classically recommended counter maneuvers, as postural sway is dynamic; thus, it may better promote pumping in the lower limbs, and could be performed proactively. Further, it can be performed in resting upright posture without a change in body position (i.e., lowering of center of mass, or reducing base of support), and could therefore be more accessible to mobility limited patients. Indeed, postural movements correspond with blood fluid volume shifts, indicating that sway may reduce orthostatic pooling associated with capillary filtration (22, 42). Additionally, in comparison to quiet (free) standing, DAP increases during supported standing, which is perhaps reflective of a reliance on vasoconstriction in the absence of skeletal muscle pumping produced from postural sway (126). However, it is unclear whether these responses are sufficiently pronounced to produce a clinically meaningful effect in syncope prevention through a role in blood pressure stability, and ultimately, increasing cerebral perfusion.

### Unconscious postural sway enhancement

**FIGURE 7:**
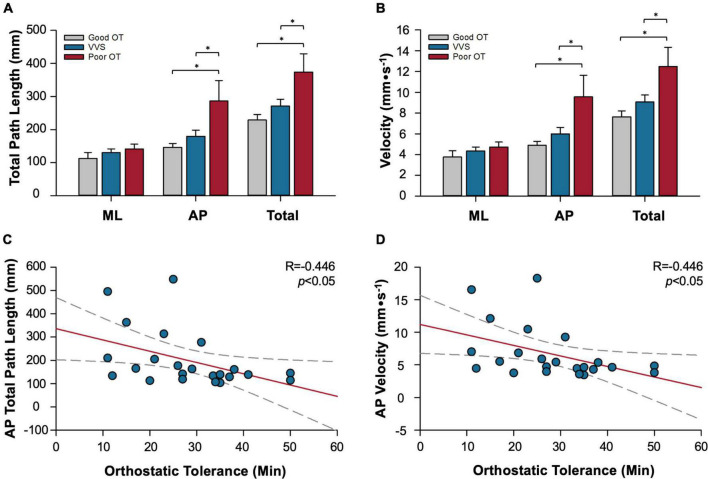
Relationships between postural sway and susceptibility to syncope. Data summarize relationships between postural sway behavior (measured as total path length or sway velocity), and cardiovascular control (measured as orthostatic tolerance, OT, time to presyncope in minutes during standard head upright tilt test with lower body negative pressure). Participant groups studied: patients with vasovagal syncope (VVS), healthy participants with good orthostatic tolerance (good OT), healthy participants with poor orthostatic tolerance (poor OT). Significant increases in the distance **(A)** and velocity **(B)** of postural sway are observed during quiet standing in individuals with poor OT who do not experience syncope in daily life, particularly in the AP direction. In patients with VVS, there is no such enhancement of postural sway to compensate for their orthostatic intolerance. The distance **(C)** and velocity **(D)** moved in the AP direction was significantly correlated with OT, where those with the lowest OT had greater postural sway. *Denotes significant difference where *p* < 0.05. ML, mediolateral component of sway; AP, anteroposterior component of sway. Adapted from Claydon and Hainsworth 2005 (111), and Claydon and Hainsworth (110).

Enhanced postural sway during quiet standing has been observed in populations with poor cardiovascular control (Figure 7). Otherwise healthy participants that did not report symptoms of syncope and presyncope in daily living, but had poor orthostatic tolerance [OT, as determined by a head-upright tilt test with lower body negative pressure (127)], demonstrated increased anteroposterior and mediolateral postural sway (quantified in terms of both path length and velocity) upon standing (114, 115). In this cohort, OT was negatively correlated with postural sway behavior (Figure 7), whereby those with the poorest orthostatic tolerance adopted greater postural sway during standing. Skeletal muscle pumping is inactivated during the head up tilt test, however, during normal standing, postural muscles are continually and reciprocally activated (125, 128). In these asymptomatic individuals with poor OT, it seems that it is this skeletal muscle pumping strategy that supplements other mechanisms of cardiovascular control to prevent presyncope and syncope in daily living.

While a relationship between postural behavior and cardiovascular control exists in this cohort, any underlying cause is still unclear. It may be that enhanced postural sway is a subconscious adaptation, or other compensatory mechanism to combat reduced cardiovascular control. Habitual leg restlessness, another discrete leg movement, has been noted in patients with orthostatic hypotension secondary to autonomic failure, and was also thought to reduce symptomatic drops in blood pressure (68). Trends of sway enhancement have been observed in other populations experiencing poor cardiovascular control such as Alzheimer’s disease (43) or deconditioned astronauts following space flight (44); increased postural sway in these populations could also be explained as a response to poor cerebral perfusion (43, 116, 129), or a simple reflection of simultaneous impairments in postural and cardiovascular control driven by the multifaceted impacts of neurodegenerative diseases or anti-gravity deconditioning. Conversely, in the case that asymptomatic individuals with poor OT are “natural swayers,” it may be that a habit of increased sway (and therefore increased muscle pumping) reduced their dependence on neural regulatory mechanisms to produce a detraining-like effect that impaired the sensitivity and efficacy of those reflexes (115).

### Postural sway and cardiovascular control

**FIGURE 8:**
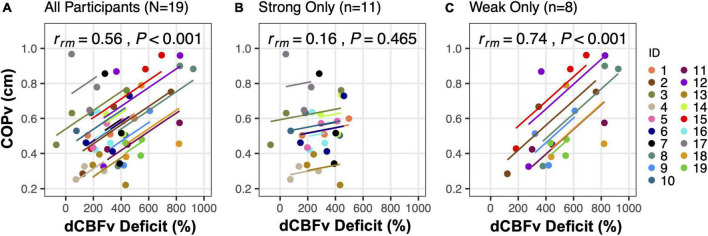
Postural sway response to cerebral hypoperfusion. Repeated measures within-participant correlations between diastolic cerebral blood flow velocity deficit (dCBFv, quantified as the difference between dCBFv in the middle cerebral artery following the supine-stand transition and its baseline value in the supine posture before any intervention) and mean center of pressure vector (COPv) are shown. Analyses were performed among **(A)** all participants, and groups of **(B)** strong regulators only, or **(C)** weak regulators only, as identified through an *a posteriori* cluster analysis (112). The number of participants (*n*) included in each condition are shown at the top of each respective plot. Parallel slopes are fitted for each participant with ANCOVA using data from the 3 postural transitions, such that the overall model error is minimized. The repeated measures correlation coefficient (*r_*rm*_)* is derived from a ratio of the sum of squares. Adapted from Fitzgibbon-Collins et al. (112).

While the contribution of locally (e.g., autocrine, paracrine) and neurally-mediated (i.e., baroreflex) responses to orthostatic stress are well understood, the regulation of skeletal muscle pumping in upright standing is less clear. Decreased blood pressure (130) and cerebral hypoperfusion (117, 131) increase postural sway in quiet standing. Similarly, during a quiet stand following a supine-stand transition, the total path length of sway increased concordantly with progressive cerebral hypoperfusion (induced by bilateral thigh cuff deflation during the stand, and hyperventilation introduced prior to the stand) (Figure 8) (116). Perhaps the increased postural sway following the postural transition results from impaired neural control that is secondary to cerebral hypoperfusion, such that impairment sets in when cerebral blood flow drops below a threshold value (116).

**FIGURE 9:**
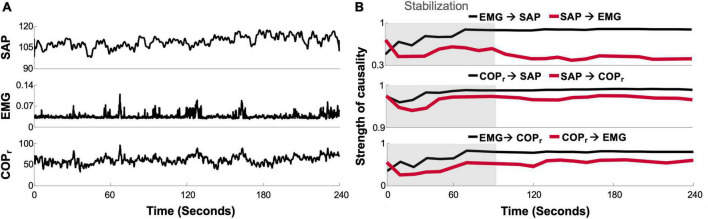
Cardio-postural causality. **(A)** Example tracing of simultaneously recorded systolic arterial pressure (SAP), calf electromyography (EMG), and center of pressure (COPr) signals. **(B)** Convergence plot of causality between signals over time during a quiet upright standing trial. Causality between signals, as calculated from the convergent cross mapping method (130, 135, 136), was measured on a scale between 0 and 1 (unitless). Gray shading indicates stabilization period following upright stance. Adapted from Verma et al. (137).

Integration between blood pressure and cardiovascular control has also been evaluated mathematically through identifying the coherence (132, 133) and causality (134) between signals of blood pressure, muscle activity, and center of pressure movements during quiet standing (Figure 9). Bidirectional coupling between fluctuations in SAP, electromyography activity, and center of pressure movement were uncovered, particularly in the period of standing after initial stabilization, such that sway was found to drive changes in SAP (presumably relating to the skeletal muscle pumping effect) but also that SAP was found to drive changes in sway (118, 119). While this provides further evidence that postural and cardiovascular systems may work together during orthostatic stress, this group also hypothesized the existence of a cardio-postural reflex whereby muscle pumping could be activated in response to hypotension (119); however, any physiological or anatomical evidence for such a mechanism is yet to be uncovered. While postural sway has been shown to increase during baroreceptor stimulation through neck suction (135), supporting an interaction between baroreflex and postural control, this stimulus simulates hypertension, and as such would be expected to be associated with a reduction in postural sway if a homeostatic mechanism between blood pressure and sway exists. Considerable evidence has also accumulated in support of a role for the vestibular system in cardiovascular control during body movement (termed the “vestibulo-sympathetic reflex”) (136), however, these are most active during pronounced postural changes, such as a sit-to-stand transition. Whether these reflexes would take effect during fine postural adjustments that occur during prolonged standing is unknown. Thus, while it is clear that the control of postural sway and cardiovascular homeostasis do overlap, the underpinnings of cause and effect, as well as our specific understanding of mechanisms, are still emerging.

## Discussion and conclusion

### Counter pressure maneuvers

CPM are a mainstay component of syncope management recommendations, and offer a cheap and relatively risk-free means to abort or delay an episode of syncope that can be adopted in addition to other commonly used preventative management strategies (e.g., avoidance measures, behavioral interventions, salt and volume loading, medication adjustment). Physiological evidence suggests that CPM hold promise in syncope prevention, with demonstrable improvements in blood pressure, SV and CBFv; however, the effects of CPM on HR, CO and TPR are more complex, and require further investigation to assess their mechanistic underpinnings, as well as to ascertain whether the significant improvements reported hold practical significance. The real-world efficacy of CPM to prevent syncope is less clear and few studies have directly assessed CPM benefit in daily living. Community-based investigations are often limited by a lack of comparator group, and inconsistent quantification of treatment success.

Ultimately, there is a need for rigorous clinical trials that include a placebo or control group in order to properly evaluate CPM efficacy. While it is recommended that patients learn multiple maneuvers so they can combine them in daily living, or select based on their social setting, abilities, or preference, comparisons between different CPM (e.g., dynamic vs. static maneuvers, movement efficacy, patient preference) would best inform clinical practice. Future trials should also include more diverse patient populations including those without a prodrome, pediatric patients, patients with POTS, and patients with cardioinhibitory VVS. These should evaluate improvement with consideration of each patient’s baseline episode frequency, presyncopal episodes that would have resolved without intervention, and improvements in patient quality of life after CPM introduction.

### Postural sway

There is emerging evidence that postural sway may contribute to the maintenance of cardiovascular homeostasis through activation of the skeletal muscle pump. Relationships between postural movements and cardiovascular responses have been identified that are suggestive of either a learned response of enhanced sway to combat orthostatic intolerance, or a more complex regulatory mechanism. While the oscillatory nature of postural sway has potential to serve as a dynamic CPM, more work is needed to identify the potential of this movement to enhance cardiovascular stability and prevent syncope.

## Author contributions

VC and EW conceived the manuscript. EW and FK conducted the search, screened articles for inclusion, and extracted data from included studies. All authors performed data analysis and visualization, interpreted the data, contributed to writing the manuscript and approved the final version for submission.
